# Fungal endophytes inhabiting mountain-cultivated ginseng (*Panax ginseng* Meyer): Diversity and biocontrol activity against ginseng pathogens

**DOI:** 10.1038/s41598-017-16181-z

**Published:** 2017-11-24

**Authors:** Young-Hwan Park, Yoosam Kim, Ratnesh Chandra Mishra, Hanhong Bae

**Affiliations:** Department of Biotechnology, Yeungnam University, Gyeongsan, Gyeongbook 38541 Republic of Korea

## Abstract

Fungal endophytes isolated from mountain-cultivated ginseng (MCG, *Panax ginseng* Meyer) were explored for their diversity and biocontrol activity against ginseng pathogens (*Alternaria panax*, *Botrytis cinerea*, *Cylindrocarpon destructans*, *Pythium* sp. and *Rhizoctonia solani*). A total of 1,300 isolates were isolated from three tissues (root, stem and leaf) from MCGs grown in 24 different geographic locations in Korea. In total, 129 different fungal isolates were authenticated by molecular identification based on internal transcribed spacer (ITS) sequences. The fungal endophytes belonged to Ascomycota (81.7%), Basidiomycota (7.08%), Zygomycota (10%) and Unknown (1.15%), with 59 genera. Analysis of diversity indices across sampling sites suggested species abundance as a function of geographical and environmental factors of the locations. Shannon diversity index and richness in the different tissues revealed that root tissues are colonized more than stem and leaf tissues, and also certain fungal endophytes are tissue specific. Assessment of the ethyl acetate extracts from 129 fungal isolates for their biocontrol activity against 5 ginseng pathogens revealed that *Trichoderma polysporum* produces the antimcriobial metabolite against all the pathogens. This result indicates the promise of its potential usage as a biocontrol agent.

## Introduction

Ginseng (*Panax ginseng* Meyer) is a perennial herb, whose roots are highly valued for medicinal purposes for more than 1,000 years^1^. The most important pharmacologically active compounds in ginseng are group of tetracyclic triterpene glycosides, known as ginsenosides, which have anticancer, antioxidant, antiplatelet and antimicrobial functions^2,3^. Ginsenosides are distributed in all part of ginseng plants with diverse profiles. Mountain-cultivated ginseng (MCG) is raised directly in forest and grown naturally to mimic mountain’s wild ginseng^4^. MCG is considered far superior than field-cultivated ginseng (FCG), with remarkable differences in ginsenoside levels and profiles^5^. Normally MCG is harvested after 10–15 years of cultivation, compared to FCG (5–6 years), during which time, plants remain vulnerable to various pathogens^6^.

Understanding the role of fungal endophytes in ecosystems has been challenged by indiscriminate pesticides and lack of characterization of fungal diversity, despite importance of symbiotic interactions between host plants and fungi^7^. The results of such symbiotic interactions can be different based on particular environmental conditions or interacting species ecology in local^8^. Therefore, it is utmost important to investigate the geographical distributions of fungal endophytes, and their ecological associations in plant populations and communities^9^. Fungal endophytes can be defined functionally as fungi that internally colonize plant tissues asymptomatically without initiating any disease^10^. Previous studies have reported that fungal endophytes may become pathogenic during senescence in their host plants or some environmental conditions^11,12^. However, most studies suggest that fungal endophytes can confer beneficial effects on their host plant: alleviation of biotic and abiotic stresses, or growth promotion^12,13^. Fungal endophytes produce numerous bioactive compounds, which are widely used in agriculture, medicine and industries manufacturing antimicrobial, insecticidal and anticancer agents^14,15^. Many studies have been conducted to survey the endophyte-plant interaction with particular emphasis on the production of secondary fungal metabolites and their potential use in biological control perspective^16,17^. Previous studies report phylogenetic diversity and biocontrol potential of fungal endophytes in field-cultivated ginseng (FCG)^18–20^, whereas fungal endophytes in MCG are still poorly characterized. Therefore, characterization of the fungal community associated with MCG is extremely important to reveal the identity of unexplored endophytes and identify novel compounds for biological control.

This study provides an overview of diversity and distribution of fungal endophytes from MCG plants. Additionally, the effect of environmental conditions on the distribution of the fungal endophytes is also revealed. Further, our investigation also brings forth the fungal endophytes that can be used in biological control of ginseng pathogens.

## Results

### Isolation and identification of fungal endophytes

**Figure 1:**
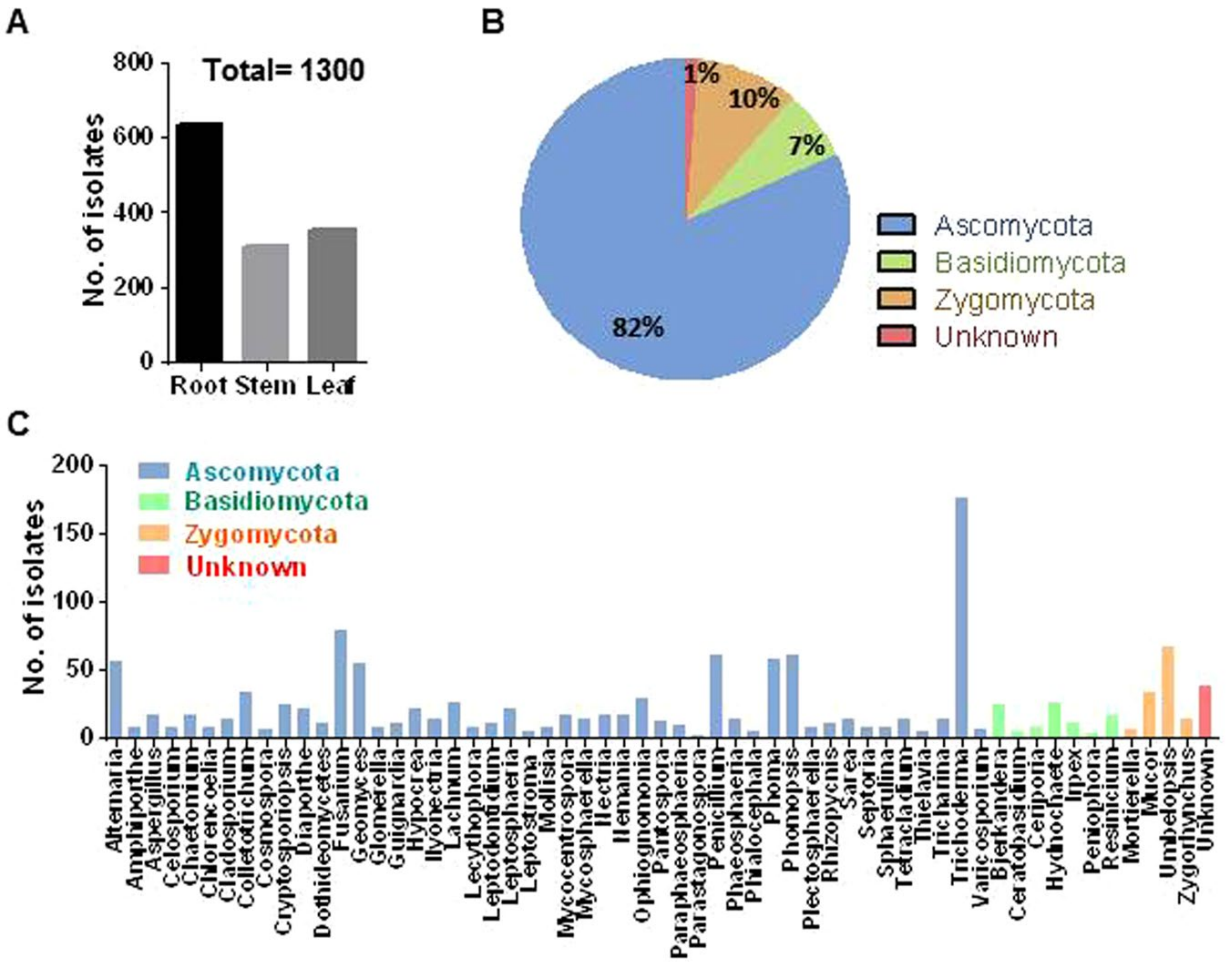
Isolation and identification of fungal endophytes from mountain-cultivated ginseng in Korea. (**A**) Bar plot showing the number of fungal isolates from root, stem and leaf tissues (total = 1,300 isolates). (**B**) Pie chart indicating the percentage area shared by different phyla. (**C**) Distribution of fungal isolates at the genus level (total = 59 genus). Different colors indicate the phyla to which each genus belongs.

**Figure 2:**
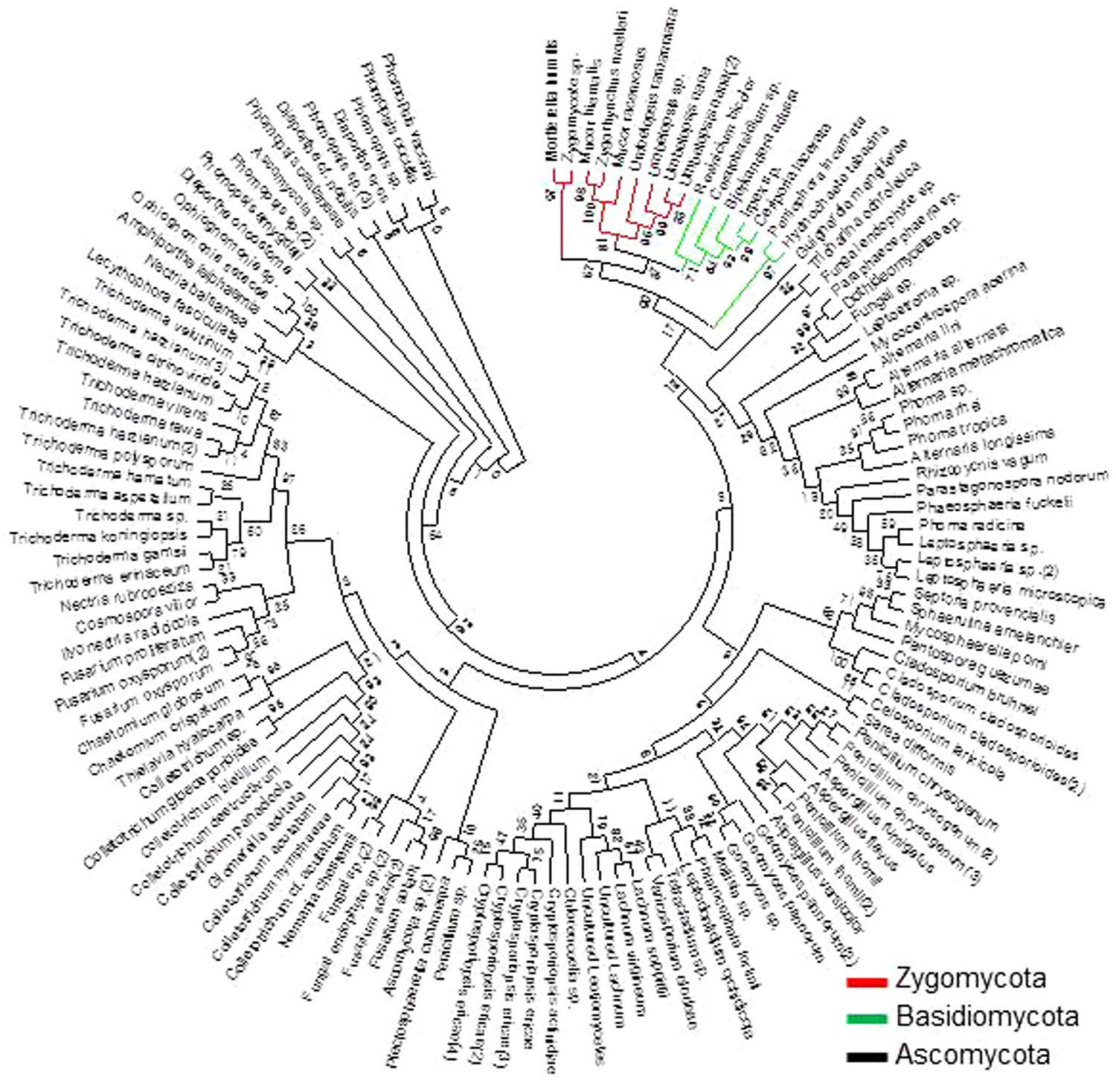
Phylogenetic tree based on neighbor-joining analysis. Tree was constructed based on the internal transcribed spacer (ITS) sequences of the fungal endophytes isolated from various tissues of mountain-cultivated ginseng. Bootstrap values expressed as percentage of 1,000 replications are indicated at the nodes.

A total of 1,300 culturable fungal endophytes were isolated from 3 different tissues of MCG plants based on morphological characteristics across 24 different sites. Root tissue hosted the largest number of fungal endophytes (637 isolates) followed by the leaf (354 isolates) and the stem tissues (309 isolates) (Fig. 1A). All isolates on pure culture were submitted for molecular identification based on ribosomal DNA internal transcribed spacer (rDNA ITS) sequence analysis. The amplified ITS sequences varied from 500 to 550 bp. These 1,300 isolates were assessed to constitute 129 isolates based on ITS sequence analysis. The detailed description of 129 fungal endophytes is listed in Supplementary Table S1. There are 4 Unknown isolates (2 fungal endophyte sp. and 2 fungal sp.). All the representative isolates were assigned to Ascomycota (81.7%), Basidiomycota (7.08%), Zygomycota (10%) and Unknown (1.15%) (Fig. 1B). Overall, 1,300 isolates were classified into 59 genera including unknown (Fig. 1C). Most of the isolates belonged to *Trichoderma* (13.6%) from phylum Ascomycota, and only seven genera (*Fusarium*, *Umbelopsis*, *Penicillium*, *Phomopsis*, *Phoma*, *Alternaria* and *Geomyces*) showed frequencies higher than 4%. The phylogenetic tree revealed the relationship between the different species of fungal endophytes (Fig. 2). The most frequently occurring Phylum, Ascomycota, included 46 genera. Basidiomycota group was represented by 7 different genera; *Bjerkandera*, *Ceratobasidium*, *Ceriporia*, *Hydnochaete*, *Irpex*, *Peniophora* and *Resinicium*. The group of Zygomycota constituted *Mortierella*, *Mucor*, *Umbelopsis* and *Zygorhynchus*. These results suggested the diversity in fungal endophytes collected from different tissues of MCGs located at different sites.

### Diversity of fungal endophytes among sites

**Figure 3:**
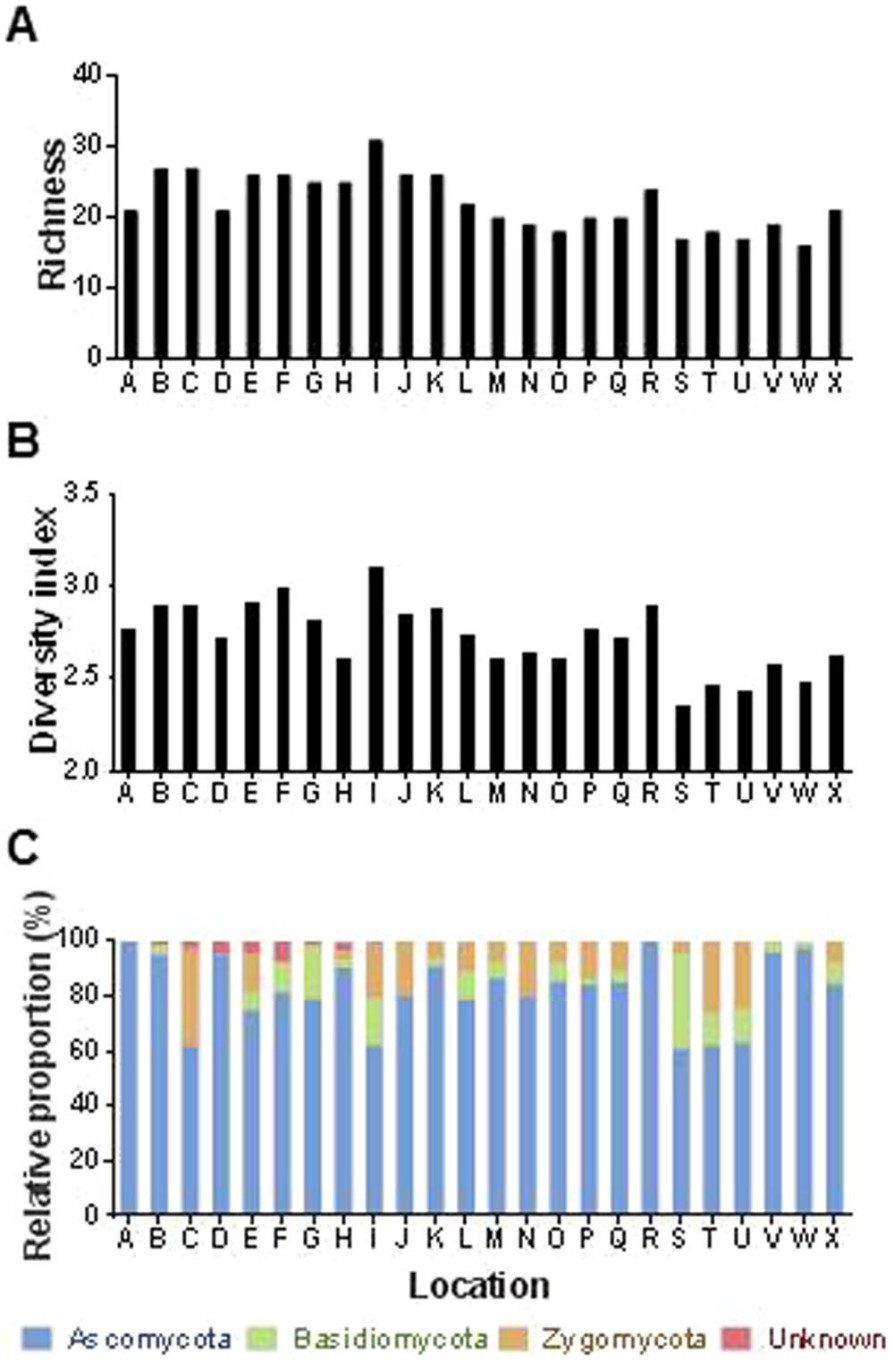
Distribution and diversity of fungal endophytes in mountain-cultivated ginseng. (**A**) Species richness across the sampling sites. (**B**) Shannon’s (H′) index across the sampling sites. (**C**) Relative proportion of fungal isolates at phylum level across sampling sites.

We found substantial variation in fungal diversity across the sampling sites (Fig. 3). Fungal diversity in a given location was significantly different from those in other locations. The species richness (S) revealed that the region I showed the richest in fungal endophytes (31 isolates), followed by the regions B and C (27 isolates), and the lowest in region W (16 isolates). Shannon’s diversity index (H) was also noted to be the highest in region I (H′ = 3.097), followed by regions F, E, R, B, C, K, J and G (H′ > 2.800), whereas it was the lowest in region S (H′ = 2.347). In relative proportion of each phylum (including Unknown), Ascomycota was found to be the most dominant phylum in all sites. Remarkably, Unknown fungal endophytes were found only in site B, C, D, E, F, G, H and I. Fungal endophytes belonging to Ascomycota were exclusively isolated in site A and R. Principal component analysis (PCA) showed the distribution of particular sites according to fungal diversity and their characteristics (Supplementary Fig. S2). The region I showed the highest Shannon diversity index and richness, and also showed the highest levels of potassium, organic matter and phosphate. There was no relation in degree of proximity among sampling sites and fungal diversity. None of the individual climatic and soil edaphic factors were explainable the association of fungal diversity across the sites based on their geographic characteristics (Supplementary Fig. S3 and Table S2).

### Diversity of fungal endophytes within tissues

**Figure 4:**
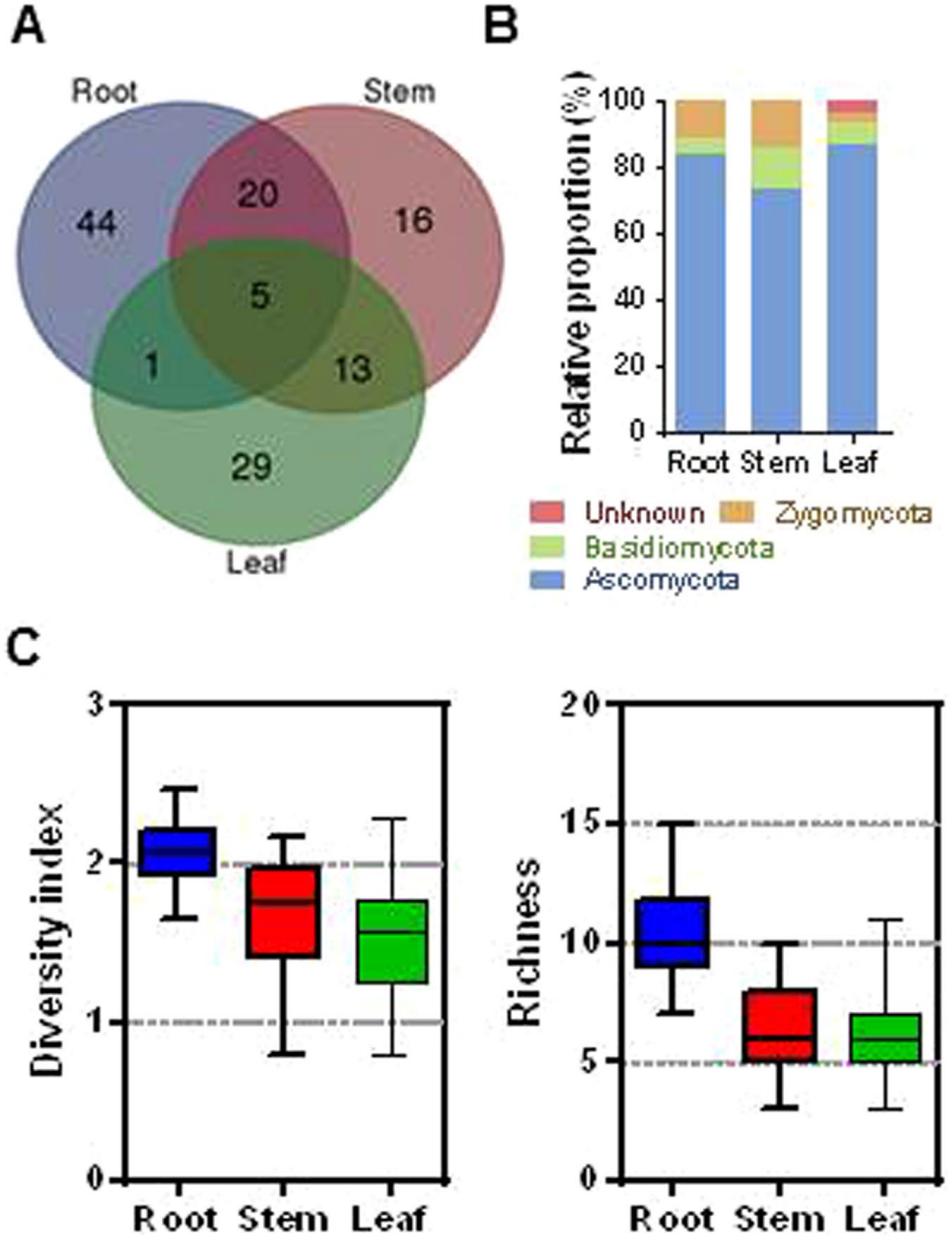
Distribution and diversity of fungal endophytes in different tissues of mountain-cultivated ginseng. (**A**) Venn diagram showing the distribution of fungal isolates recovered from root, stem and leaf tissues. (**B**) Relative proportion of fungal endophytes in different tissues at phylum level. (**C**) Boxplots showing species richness (left) and Shannon’s (H′) index (right) in root, stem and leaf tissues. Different letters on the each bar indicated difference at 5% level (*P* < 0.05) of significance by Duncan’s multiple range tests.

The highest number of different fungal endophytes was identified in root tissues (70 isolates) followed by the stem (54 isolates) and the leaf (48 isolates) (Fig. 4). Almost 70% of isolates (44, 16 and 19 isolates in root, stem and leaf, respectively) were observed to be specific for a particular tissue, while 5 isolates (*Alternaria longissima*, *Trichoderma erinaceum*, *Trichoderma harzianum* and *Umbelopsis nana*) were found in all 3 tissues. The relative proportion of fungal endophytes in tissues showed that Ascomycota is the most abundant phylum. Notably, isolates belonging to Unknown were present only in leaf tissues. Diversity of endophytes measured by Shannon diversity index (H′) was noted to be the highest in root tissues (*P* < 0.0001). The species richness in root tissues was also significantly higher than in stem and leaf tissues (*P* < 0.0002).

### Antimicrobial activity of ethyl acetate extracts of fungal endophytes

**Figure 5:**
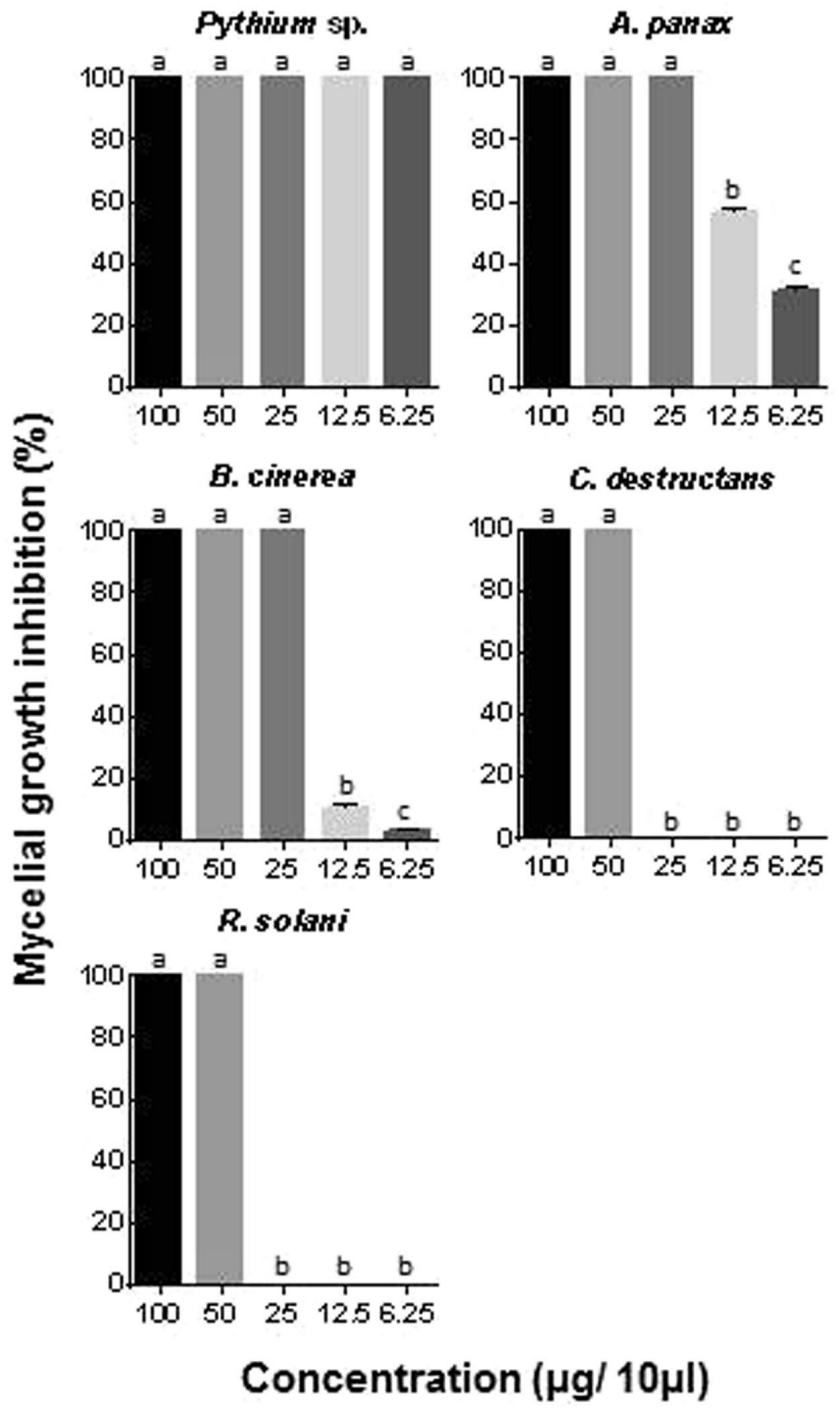
Minimum inhibitory concentration determination using ethyl acetate extracts of *Trichoderma polysprum* against ginseng pathogens. Mycelial growth inhibition (%) = [(mycelial growth of control − mycelial growth of treatment)/mycelial growth of control] × 100. Bars represent the mean and standard error of 2 experiments with 3 biological replications. Different letters in the same column indicated difference at 5% level (*P* < 0.05) of significance by Duncan’s multiple range tests.

A total of 129 fungal endophytes were isolated from various tissues of MCGs, and their crude ethyl acetate extracts (100 μg/10 μl) were used in the biocontrol assay to investigate antimicrobial activity against 5 different ginseng pathogens. Among them, 7 fungal isolates (*Tricharina ochroleuca*, *Trichoderma polysporum*, *Lachnum virgineum*, *Phoma* sp., *Alternaria longissimi*, Fungal endophyte sp. and *Penicillium chrysogenum*) effectively inhibited pathogen growth in preliminary screening, using modified agar diffusion method (Table 1). Importantly, ethyl acetate extract of *T*. *polysporum* showed the highest activity against all pathogens with 100% mycelial growth inhibition. Second best activity was reported with *P*. *chrysogenum*, showing 100% inhibition, except in *A*. *panax* (73%). Five different concentrations (100, 50, 25, 12.5 and 6.25 μg in 10 μl) of ethyl acetate extract of *T*. *polysporum* were tested to determine minimum inhibitory concentration (MIC). *Pythium* sp. was the most sensitive to the extract showing 100% mycelial growth inhibition in all concentrations (Fig. 5). The concentration of 25 μg was determined as MIC against *A*. *panax* and *B*. *cinerea*, whereas 50 μg was exhibited as MIC against *R*. *solani* and *C*. *destructans*.

**Table 1:** Effect of ethyl acetate extracts of different fungal endophytes isolated from mountain-cultivated ginseng on mycelial growth of ginseng pathogens.

Endophyte	*B*. *cinerea*	*R*. *solani*	*A*. *panax*	*C*. *destruntans*	*Pythium* sp.
	Mycelial growth inhibition (%)				
*Tricharina ochroleuca*	100^a^	100^a^	100^a^	95.3 ± 1.3^b^	55.8 ± 0.9^c^
*Trichoderma polysporum*	100^a^	100^a^	100^a^	100^a^	100^a^
*Lachnum virgineum*	100^a^	25.5 ± 1.1^c^	73.0 ± 1.5^ab^	100^a^	100^a^
*Phoma* sp.	100^a^	81.1 ± 2.2^ab^	100^a^	100^a^	100^a^
*Alternaria longissima*	50.0 ± 1.3^c^	78.8 ± 1.1^ab^	77.3 ± 1.3^b^	0^c^	45.0 ± 2.3^d^
Fungal endophyte sp.	53.3 ± 3.3^b^	74.3 ± 1.2^b^	68.3 ± 1.6^b^	100^a^	72.2 ± 1.9^b^
*Penicillium chrysogenum*	100^a^	100^a^	73.0 ± 1.5^b^	100^a^	100^a^

Crude ethyl acetate extracts (100 μg/10 μl) were used in the biocontrol assay. *Values are given as the means (standard error) of three replications. Different letters in the same column indicated difference at 5% level (*P* < 0.05) of significance by Duncan’s multiple range tests.

### Antagonistic activity of *T*. *polysporum*

**Figure 6:**
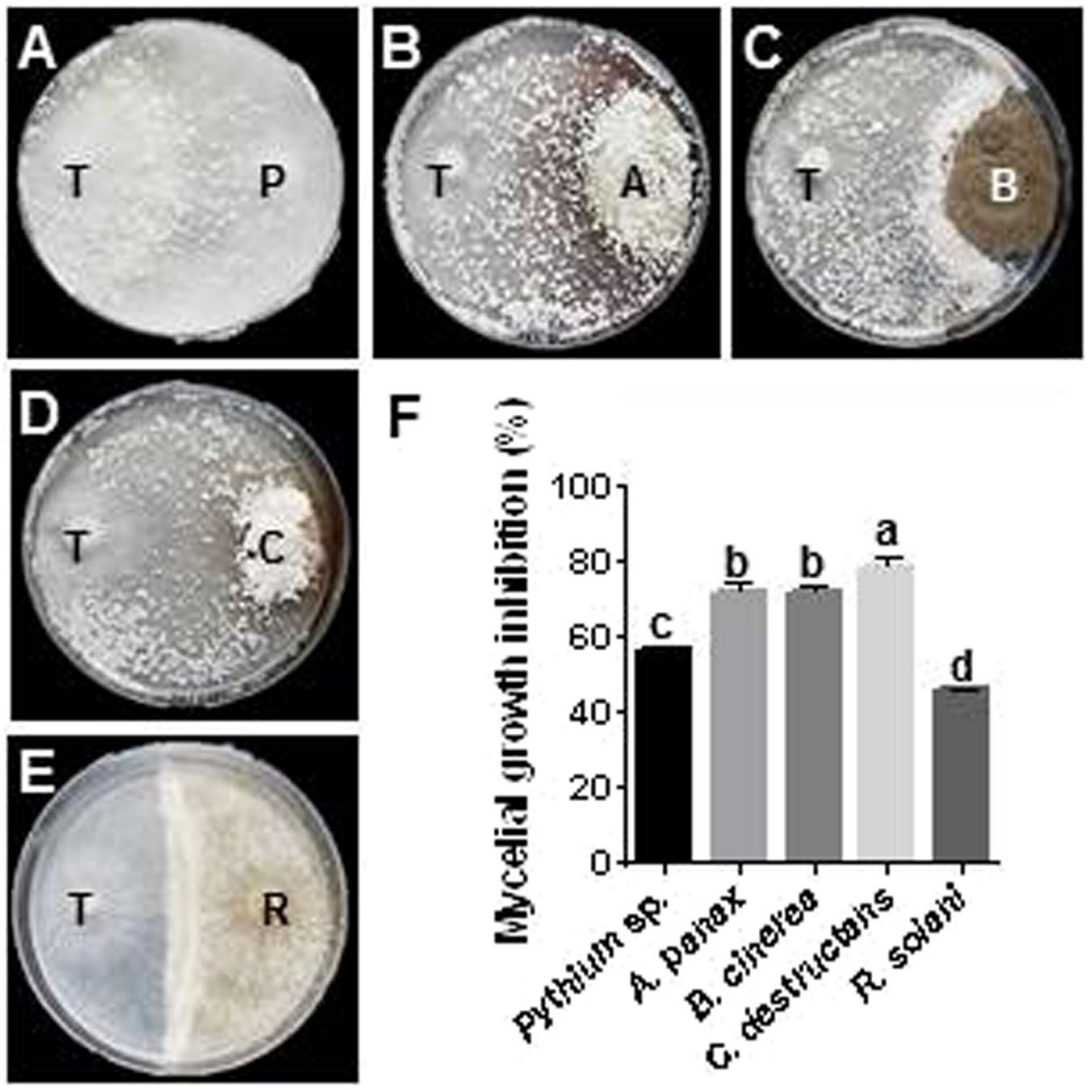
Interactions of *Trichoderma polysprum* and ginseng pathogens observed in dual culture antagonistic assay. (**A**) through (**E**), where endophyte *T*. *polysprum* (T) placed on the left and ginseng pathogen [*Pythium* sp. (P), *A*. *panax* (**A**), *B*. *cinerea* (**B**), *C*. *destruntans* (**C**) and *R*. *solani* (R)] on the right of the representative plates. (**F**) Mycelial growth inhibition (%) by *T*. *polysprum*. Bars indicate the mean and standard error of 2 experiments with 3 biological replications. Different letters on the each bar indicated difference at 5% level (*P* < 0.05) of significance by Duncan’s multiple range tests.

*T*. *polysporum* showed significant inhibitory activity against myceilial growth of ginseng pathogens in dual culture (45.6–78.6%) (Fig. 6). The highest inhibitory effect was seen on the mycelial growth of *C*. *destructans*, with a reduction of 78.6% compared to the control. The mycelial growth of *Pythium* sp., *A*. *panax* and *B*. *cinerea* showed 55–70% inhibition, while the lowest inhibition was detected in *R*. *solani* (45.6%). *T*. *polysporum* was overgrown against *Pythium* sp., *A*. *panax* and *C*. *destructans* with profuse sporulation, which rapidly colonized the complete plate. Although no overgrowth of *T*. *polysporum* was observed against *B*. *cinerea* and *R*. *solani*, the growth of the pathogen was completely stopped at physical contact area with or without sporulation.

## Discussion

The last few decades have seen endophytes as valuable natural resources with diverse utilization in a variety of areas such as agriculture and biotechnology^21,22^. Recently, many reports have demonstrated the importance of endophytes in plant disease control^23,24^. Although the interest in *P*. *ginseng* as a medicinal plant is increasing in medical and chemical fields with regard to its active ingredients in particular ginsenoside, there are only a few investigations about its endophytic community. Moreover, the interaction between the endophytic community and ginseng plant in the light of its ecological significance is poorly studied and remained to be extensively exploited. Herein, we report the isolation and identification of fungal endophytes colonizing different tissues of MCGs, which were collected from 24 different sites in Korea. We used the culture-dependent approach to investigate the distribution and diversity of fungal endophytes in MCG. In addition, we isolated the endophytes that produce antimicrobial compounds, which can resist pathogen attack in MCG cultivation.

A total of 1,300 fungal isolates were obtained from 3 different tissues of MCGs collected from 24 different sites. The morphological characteristics and ITS sequencing showed that fungal endophytes comprise 129 isolates belonging to 3 phylum, including Unknown, with 59 different genera. Among these, 10 genera (*Alternaria*, *Nemania*, *Phoma*, *Colletotrichum*, *Phomopsis*, *Trichoderma*, *Fusarium*, *Cladosporium*, *Nectria* and *Leptodontidium*) were already isolated as endophytes from FCG in our previous studies^25,26^, but most of them were not reported previously. This result thus suggests that fungal community of MCG is more diverse than FCG. It may be because the use of pesticides was strictly prohibited in MCG cultivation. The majority of the fungal endophytes isolated from MCG belonged to filamentous Ascomycota; this phylum is one of the most diverse phyla of eukaryotes and comprises more than 3,000 genera of plant pathogens^27^. Interestingly, many of the identified fungal endophytes were plant pathogens, such as *Colletotrichum* spp, *Fusarium* spp, *Phoma* spp, *Alternaria* spp and *Diaporthe* spp. Previous studies suggest that the physical encounter between a plant and a fungus is a decisive factor in establishing a plant-endophyte association. It provides opportunity to a particular fungus to establish itself as an endophytes^28^. Pathogenic-endophytic lifestyles are interchangeable, depending on the various environmental, chemical and/or molecular triggers^23^. Thus, it is possible that a particular fungi living as an endophyte in host plant in one ecosystem can turn pathogenic in another ecological niche. *Phoma terrestris*, which was isolated as endophyte from FCG, is known as a phytopathogen that causes pink root in onion^20^. *P*. *terrestris* produces N-amino-3-hydroxy-6-methoxyphthalimide and 5H-dibenz [B,F] azepine, which show strong antimicrobial activity against ginseng pathogens^20^.

The plant-endophyte association largely depends on hosts and environmental factors, including plant genotypes, geographic locations, and edaphic factors^24^. A large diversity in fungal endophytes was observed in MCGs among the sampling sites, warranting the determination of the factors that could possibly explain this diverse composition and distribution. However, we could not find any factor such as geographical location, climate, and chemical composition of soil that might have acted as a pressing force in framing fungal endophyte communities in MCGs. This suggests that endemism together with random nature of endosphere colonization are likely community-shaping force functioning for fungal endophytes in MCG. Limitation in dispersion is likely the cause for such endemism. Additionally, the location of MCG growing sites at remote places with little disturbances corroborates this hypothesis further. Nevertheless, it is important to note that assessments of edaphic and climatic factors might not represent the precise environmental conditions of individual sampling sites. Another thing to note is the unavailability of genotype data, although the genetic variations among MCGs are generally assumed to be little. Although the above mentioned factors had no or little correlation to variation in fungal endophyte diversity, some of the individual factors might be able to account for the observed changes in some fungal endophytes across the sites. For instance, *Trichoderma*, which was the most abundant genera, was isolated from sites of Gangwon (54.5%, 96 isolates in B, F, G, H, I and L). The sites in Gangwon are at high altitude (399 m) and have high available phosphate (360 mg/kg) as compared with mean value of 24 different sites. We confirmed the positive correlations between *Trichoderma* and individual factors with regression analysis using ANOVA (*P* value < 0.005 and 0.02, respectively) (data not shown). In contrast, genera of *Geomyces* (4.2% frequency) was consistently isolated from the sites with low level of available phosphate, with just 5.45% of *Geomyces* in sites of Gangwon (*P* value < 0.003). As Gangwon province is the largest forest area in Korea, the high availability of wood in the respective MCG cultivation sites is another possible explanation for the abundance of *Trichoderma* as wood degraders and endophytes in these sites. The evidence presented is consistent with other studies stressing on the high variability of the genus members in forest areas^29^. Corroboratively, *Trichoderma* spp. have been well-known for the biodegradation of plant debris, wood and bark.

We brought forth that a wide range of fungal isolates from 59 different taxonomic genus exist as endophytes in root, stem and leaf tissues of MCG. Some endophytes showed preference for specific tissues; 44 isolates were only isolated from root whereas 16 and 29 isolates were isolated from stem and leaf, respectively. There were only 5 isolates in common between these 3 plant tissues. Tissue specificity of a particular endophyte suggests that certain species perpetuate within the specific chemistry or texture of a particular tissues, due to the differences in the anatomical structure and physiological conditions^19^. Furthermore, the diversity and species richness of fungal endophytes were higher in root than stem and leaf tissues. Soil-borne fungi are commonly more prevalent and diversified than those that infect aerial plant tissues, and ginseng roots are suitable for the growth of endophytes since it has abundant nutrients^18^. We also observed an unequal distribution of isolate richness among species with relative abundance of each endophyte identified in MCG. For example, 5 isolates, which belongs to *Alternaria*, *Trichoderma* and *Umbelopsis* that were common in all tissues, constituted 6.3% of total fungal isolates in this study. Similar results have been previously reported in other plants^30,31^.

We hypothesized that the fungal endophytes may prove beneficial in strategizing effective approaches to control ginseng pathogens and help the host survival against pathogen attack during plantation. Thus, ethyl acetate extracts of fungal endophytes were tested for antimicrobial activity against major ginseng pathogens. Indeed, seven isolates effectively inhibited pathogen growth in preliminary screening. The crude extract of *T*. *polysporum* showed the strongest inhibitory activity against all ginseng pathogens, implicating that this isolate produces the most effective antimicrobial compounds. Other isolates showed selective activity against one or more pathogens. Crude extract of *T*. *polysporum* exhibited effective inhibitory activity against *Pythium* sp., *A*. *panax*, *B*. *cinerea*, *R*. *solani* and *C*. *destructans* with the MIC of 6.25, 25, 25, 50 and 50 μg in 10 μl, respectively. Interestingly, several *Trichoderma* spp. were already used in agriculture as biocontrol agents against fungal diseases in plants. Either they produce active antimicrobial compounds and/or compete for nutrients through mycoparasitism^32–34^. Corroboratively, we also observed high antagonistic behavior in all endophyte-pathogen interactions in dual culture assay. Additionally, *T*. *polysporum* has already been reported to have antimicrobial activities against various pathogens^35,36^. We recently reported that fungal endophytes from FCG (*P*. *terrestris*) could strongly inhibit the growth of ginseng pathogens by producing antimicrobial metabolites^20^. This result also showed the great potential of fungal endophytes in biocontrol of ginseng pathogens.

Taken together, our results revealed that diverse fungal endophytes dwelling in different tissues of MCG are not influenced by biogeography of the host species. Remarkably, the community of fungal endophytes is different at all sites and tissues. The fungal endophytes keep great promise not only as biocontrol agents, but also as resources of biologically active novel metabolites. In present study, metabolite produced by *T*. *polysporum* has exhibited promising antimicrobial activity against ginseng pathogens. A complete understanding of complex plant-microbe interactions will be helpful in selecting the promising pesticide or fertilizer precisely, and other approaches for better cultivation of ginseng plant.

## Materials and Methods

### Collection of mountain-cultivated ginseng (MCG) plants

To maximize the chance of obtaining various fungal endophytes, we collected 4-year-old MCGs (*Panax ginseng* Meyer) from undisturbed mountain areas of 24 different sites in Republic of Korea during the growing season, June 2013 (Supplementary Table S1 and Fig. S1). Ten healthy plants from each site served as replicates. The harvested plants were preserved at 4 °C and plant samples were processed for isolation of fungal endophytes immediately within 24 h after harvest.

### Isolation of fungal endophytes

Fungal endophytes were isolated following previously established procedures^19^. Ginseng plants were washed thoroughly with running tap water for 5 min. The root and stem samples were cut into 10 × 10 mm segments, and the leaves were cut into 5 × 5 mm segments using a sterilized blade. Collected tissues were surface sterilized in 70% ethanol solution for 30 sec, transferred to a 2% solution of sodium hypochlorite for 10 min, and then washed with 70% ethanol for 30 sec, followed by 3 rinses in sterile distilled water for 1 min. The surface-sterilized samples were allowed to dry on sterile paper towels. After drying, six to seven tissue segments were placed on a petri dish containing potato dextrose agar (PDA) with 200 mg/L ampicillin and streptomycin to inhibit bacterial growth and then incubated at room temperature for 2 to 3 w^37^. The tissue segments were observed every day for the appearance of fungal growth. The fungal mycelia growing out of the tissue segments were collected from the culture plate and continuously maintained on fresh PDA plates. The fungal isolates were numbered and 10% glycerol stocks were made for long-term storage of fungal endophytes at −80 °C.

### Identification of fungal endophytes

For the isolation of total genomic DNA of fungal endophytes, mycelia was ground in liquid nitrogen and the powder was processed using the DNeasy Plant Mini Kit (Qiagen, CA, USA), according to manufacturers’ protocol. The universal primers, ITS1 (5′-TCCGTAGGTGAACCTGCGG-3′) and ITS4 (5′-TCCTCCGCTTATTGATATGC-3′) were used to amplify the internal transcribed spacer (ITS) region. The PCR reaction mixture (50 µl) contained 50–150 ng of DNA, 200 ρmol of each primer, 100 ρmol of each dNTP, 1.25 U *Taq* DNA polymerase and 5 µl PCR buffer. The following PCR reactions were performed: Initial denaturation at 94 °C for 3 min, followed by 30 cycles of 94 °C for 30 sec, 55 °C for 30 sec, and 72 °C for 1 min, and a final extension at 72 °C for 7 min. The PCR products were run on a 1.6% agarose gel and the DNA band was excised from the gel. PCR products were sent to COSMO Genetech (Seoul, Republic of Korea) for sequencing. All ITS sequences were analyzed through BLAST search in the NCBI database. The phylogenetic tree was constructed using neighbor-joining method and evaluated by bootstrap analysis with 1,000 replications by MEGA6^38^.

### Antimicrobial activity test of ethyl acetate extracts from fungal endophytes

After isolation of fungal endophytes, their ethyl acetate extracts were prepared and tested for antimicrobial activity against major ginseng pathogens. Ginseng pathogens were procured from Rural Development Administration Genebank Information Center (RDA GIC) (Suwon, Republic of Korea): *Alternaria panax* KACC42461 (Korea Agricultural Culture Collection), *Botrytis cinerea* KACC43521, *Cylindrocarpon destructans* KACC44656, *Pythium* sp. KACC40581 and *Rhizoctonia solani* KACC40123. Five plugs of fungal endophytes grown on PDA were inoculated into 50 ml of potato dextrose broth (PDB) in 100 ml Erlenmeyer flask. After 7 d of growth at 25 °C and 150 rpm, the equal volume (50 mL) of ethyl acetate was added into the flask and mixed well using shaker for 30 min at 100 rpm. After being allowed to stand for 1 h, top clear phase was transferred to a round flask and concentrated in a rotatory evaporator at 60 °C. After complete evaporation, the crude ethyl acetate extract was obtained and dissolved in methanol with 1% DMSO. The modified agar diffusion method^39^ was used to determine the antimicrobial activity of the ethyl acetate extracts against 5 ginseng pathogens. Six plugs of pathogen were placed equidistantly in a PDA plate. Subsequently, sterile filter paper discs (0.6-cm diameter) containing ethyl acetate extracts (100 μg/10 μl) from fungal endophytes were placed on the top of each plug. The filter paper disc containing the same solvent (methanol with 1% DMSO) was used as control. The plates were then incubated at 25 °C for 2–7 d. The growth diameter of pathogens was measured and growth inhibition (%) was calculated using the following equation: growth inhibition (%) = [growth of control − growth of treatment)/growth of control] × 100. Experiments were conducted with 3 biological replicates.

### Determination of minimum inhibitory concentration (MIC)

To determine the MIC, different concentrations of ethyl acetate extract of *T*. *polysporum* were tested against ginseng pathogens using the method described above. Six agar plugs of each pathogen were placed in the middle of 6-well plate containing PDA. Sterile filter paper discs (0.6-cm diameter) were placed on the top of each plug containing pathogen. The filter paper discs contained different concentration of ethyl acetate extracts (100, 50, 25, 12.5 and 6.25 μg in 10 µl methanol with 1% DMSO) and the solvent (10 µl methanol with 1% DMSO) as control. The 6-well plates were incubated at 25 °C for 2–7 d and colony diameter was measured daily. The experiment was repeated 3 times with 3 biological replicates.

### Dual culture bioassay

After the above antimicrobial activity test, dual culture bioassay was conducted. Herein, each of the 5 ginseng pathogens was allowed to grow together with *T*. *polysporum*. Agar plugs (0.5-cm diameter) of both *T*. *polysporum* and ginseng pathogens were co-cultured in PDA plate (4-cm apart) and the plate was incubated for 5–6 d at 25 °C. The pathogens alone were inoculated as controls. Relative growth inhibitions (%) were calculated against the control plates using the equation: growth inhibition (%) = [growth of control − growth of treatment)/growth of control] × 100. Experiments were conducted with 3 sets of replication plates.

### Data analysis

Relative proportion was calculated as a percentage of the number of isolates belonging to species or phylum divided by the total isolates recovered from all the tissue samples. Species richness (S) among the fungal endophytes was estimated based on the particular site or tissue. The Shannon’s diversity index (H) was calculated using the following equation: H = −Σ(pi × ln pi) where pi is the relative proportion of species i in a particular tissue or site^40^. Principal component analysis (PCA) and correlation analysis were conducted using R statistics software v3.4.0^31^. PCA indicates the interrelationships between fungal endophytes recovered from particular tissue and site, while correlation analysis shows the concurrence between fungal diversity with geographical and edaphic factors in a particular site. Duncan’s multiple range test (DMRT) was performed to calculate the significant differences among the values at a level of *P* = 0.05 using SAS v 9.4 (NC, USA). Graphs were made using a statistical software GraphPad Prism 6 project (CA, USA).

## Electronic supplementary material

Supplementary Figures and Tables
