# Supplementary material for: Fungal endophytes inhabiting mountain-cultivated ginseng (Panax ginseng Meyer): Diversity and biocontrol activity against ginseng pathogens
Source: Sci Rep. 2017 Nov 24;7:16221. doi: 10.1038/s41598-017-16181-z (PMC5701219; doi:10.1038/s41598-017-16181-z)
Supplement: Supplementary file 1 — Supplementary Figures and Tables [file 41598_2017_16181_MOESM1_ESM.pdf]

Fungal endophytes inhabiting mountain-cultivated ginseng (*Panax ginseng* Meyer): Diversity and biocontrol activity against ginseng pathogens

Young-Hwan Park, Yoosam Kim, Ratnesh Chandra Mishra and Hanhong Bae*

Department of Biotechnology, Yeungnam University, Gyeongsan, Gyeongbook 38541, Republic of Korea

*Correspondence :

Hanhong Bae

Professor, Department of Biotechnology, Yeungnam University, Gyeongsan 38541, Republic of Korea, Phone: 8253-810-3031 (office), Fax: 8253-810-4769, Email: hanhongbae@ynu.ac.kr

Keywords : fungal endophyte, mountain-cultivated ginseng, fungal diversity, biological control, ethyl acetate extract

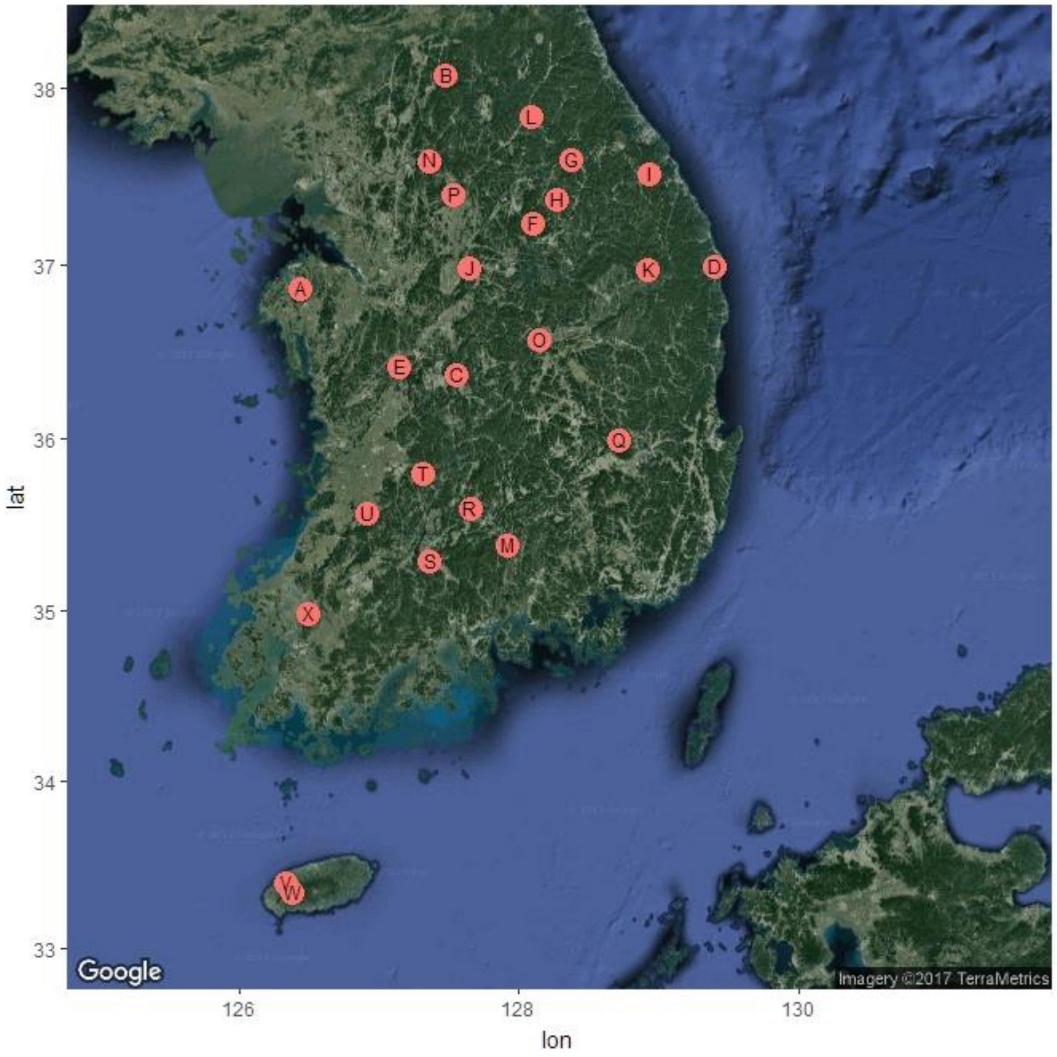

Supplementary Figire S1. A map of Republic of Korea showing locations of 24 sampling sites. Each circle indicates different sampling site labeled as A to X. Google map was used to represent sampling sites using ggmap package (Kahle, D. & Wickham, H. ggmap: Spatial visualization with ggplot2. *R J* **5**, 144–161) in R program (http://www.R-project.org).

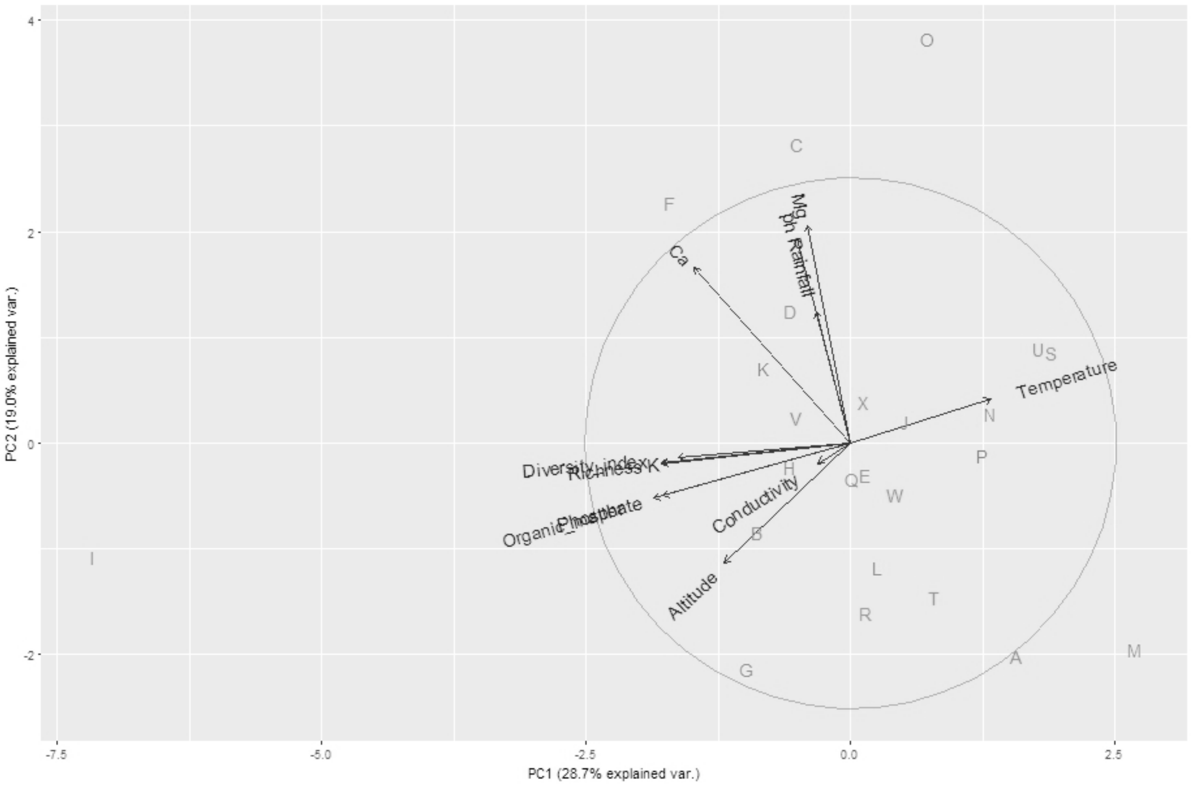

Supplementary Figure S2. Principal component analysis (PCA) of variability of fungal endophyte communities based on 24 different geographic locations with their climatic and soil edaphic factors. PCA was conducted using R software ver.3.4.0.

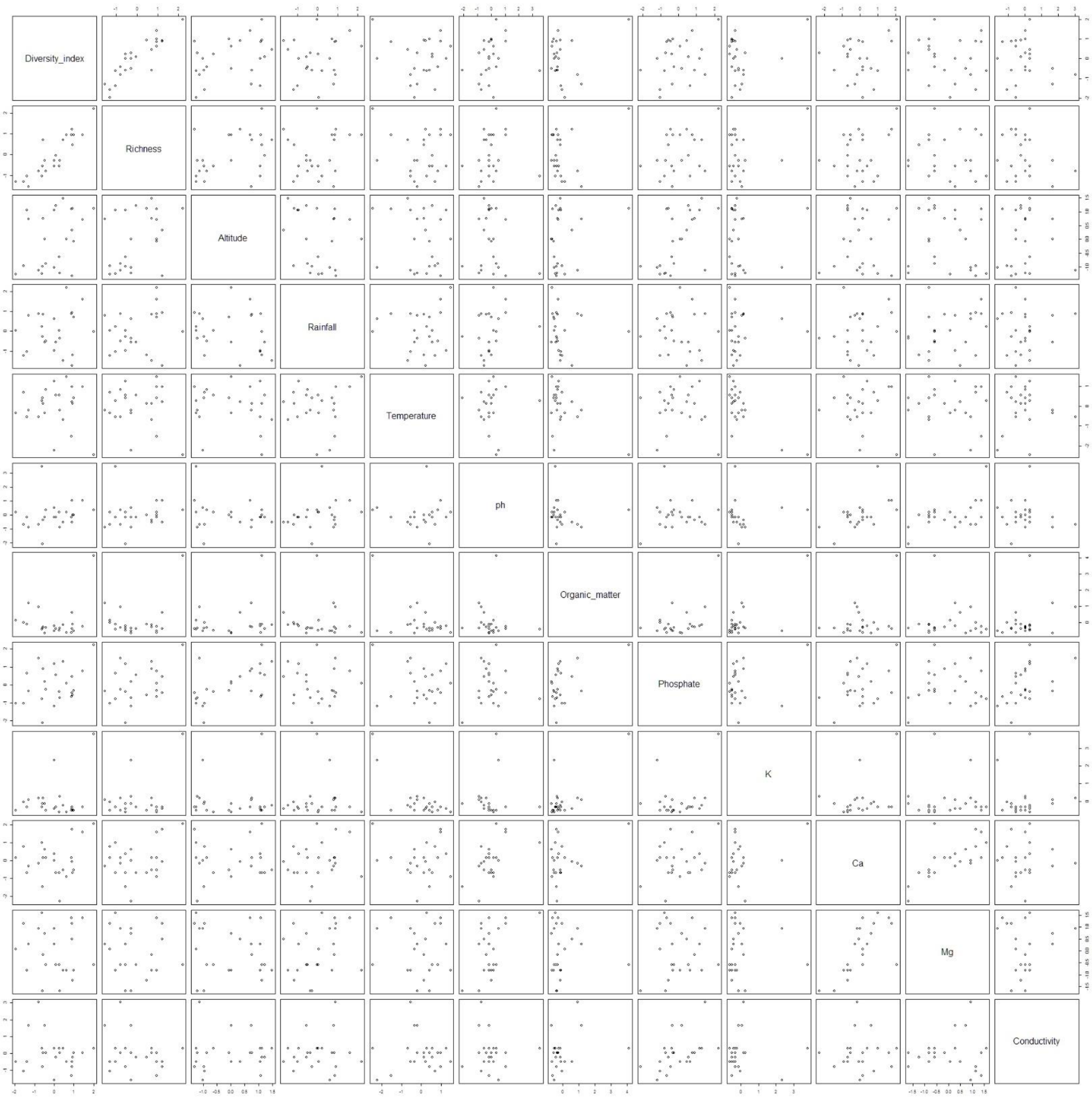

Supplementary Figure S3. Scatter plots showing relationships between meta-data of sampling sites with their Shannon's index and species richness performed using R software ver. 3.4.0.

Supplementary Table S1. Molecular identification of fungal endophytes isolated from Panax ginseng Mayer based on ITS rDNA analysis.

No.	Putative taxon	Accession No.	Phylum	% Similarity	No.	Putative taxon	Accession No.	Phylum	% Similarity
1	*Alternaria alternata*	KF438091.1	Ascomycota	100	66	Leptostroma sp.	KF574888.1	Ascomycota	100
2	*Alternaria lini*	KF293969.1	Ascomycota	99	67	*Mollisia* sp.	DQ069036.1	Ascomycota	100
3	*Alternaria longissima*	KU310611.1	Ascomycota	99	68	*Mortierella humilis*	JX976125.1	Zygomycota	100
4	*Alternaria metachromatica*	KF496083.1	Ascomycota	98	69	*Mucor hiemalis*	KC888987.1	Zygomycota	100
5	*Amphiporthe leiphaemia*	AJ293882.1	Ascomycota	99	70	*Mucor racemosus*	JN205989.1	Zygomycota	97
6	Ascomycota sp.	FJ375156.1	Ascomycota	97	71	*Mycocentrospora acerina*	KC585407.1	Ascomycota	98
7	Ascomycota sp.	HQ608112.1	Ascomycota	100	72	*Mycosphaerella pomi*	AB795263.1	Ascomycota	100
8	*Aspergillus flavus*	KF562205.1	Ascomycota	100	73	*Nectria balsamea*	HM484540.1	Ascomycota	100
9	*Aspergillus fumigatus*	EF567970.1	Ascomycota	100	74	*Nectria rubropeziza*	HQ897797.1	Ascomycota	100
10	*Aspergillus versicolor*	KC461554.1	Ascomycota	100	75	*Nemania chestersii*	AJ390430.1	Ascomycota	100
11	*Bjerkandera adusta*	KF475891.1	Basidiomycota	100	76	*Ophiognomonia setacea*	JQ414227.1	Ascomycota	100
12	*Celosporium larixicola*	FJ997287.1	Ascomycota	100	77	*Ophiognomonia* sp.	JQ414285.1	Ascomycota	96
13	*Ceratobasidium* sp.	GU937738.1	Basidiomycota	99	78	*Pantospora guazumae*	JN190956.0	Ascomycota	100
14	*Ceriporia lacerata*	KC881071.1	Basidiomycota	97	79	*Paraphaeosphaeria* sp.	KJ469553.1	Ascomycota	100
15	*Chaetomium crispatum*	JX280781.1	Ascomycota	100	80	*Parastagonospora nodorum*	KF251177.1	Ascomycota	100
16	*Chaetomium globosum*	KF305748.1	Ascomycota	100	81	*Penicillium chrysogenum*	DQ249212.1	Ascomycota	100
17	*Chlorencoelia* sp.	JQ754001.1	Ascomycota	95	82	*Penicillium chrysogenum*	JN585943.1	Ascomycota	100
18	*Cladosporium bruhnei*	KC461496.1	Ascomycota	100	83	*Penicillium* sp.	AY354257	Ascomycota	99
19	*Cladosporium cladosporioi*	KC464337.1	Ascomycota	100	84	*Penicillium thomii*	JQ034359.1	Ascomycota	100
20	*Cladosporium cladosporioi*	KF278646.1	Ascomycota	100	85	*Penicillium thomii*	JX535130.1	Ascomycota	100
21	*Colletotrichum acutatum*	KC916710.1	Ascomycota	100	86	*Peniophora incarnata*	KP050580.1	Basidiomycot	100
22	*Colletotrichum bletillum*	JX625178.1	Ascomycota	99	87	*Phaeosphaeria fuckelii*	EF151447.1	Ascomycota	100
23	*Colletotrichum cf. acutatum*	HM172824.1	Ascomycota	99	88	*Phialocephala fortinii*	HQ406812.1	Ascomycota	100
24	*Colletotrichum destructivum*	JX625174.1	Ascomycota	100	89	*Phoma radicina*	JQ676200.1	Ascomycota	100
25	*Colletotrichum gloeosporio*	JQ814338.1	Ascomycota	100	90	*Phoma rhei*	KF531831.1	Ascomycota	100
26	*Colletotrichum nymphaeae*	KC840354.1	Ascomycota	98	91	*Phoma* sp.	KC456276.1	Ascomycota	100
27	*Colletotrichum panacicola*	GU935869.1	Ascomycota	100	92	*Phoma tropica*	JQ954396.1	Ascomycota	100
28	*Colletotrichum* sp.	EF608062	Ascomycota	95	93	*Phomopsis amygdali*	JX182974.1	Ascomycota	99
29	*Cosmospora vilior*	HM061314.1	Ascomycota	100	94	*Phomopsis castaneae*	JF957786.2	Ascomycota	100
30	*Cryptosporiopsis actinidiae*	AY359234.1	Ascomycota	99	95	*Phomopsis occulta*	HM439635.1	Ascomycota	100
31	*Cryptosporiopsis ericae*	JX406516.1	Ascomycota	99	96	*Phomopsis* sp.	FJ176469.1	Ascomycota	99
32	*Cryptosporiopsis ericae*	GU934585.1	Ascomycota	99	97	*Phomopsis* sp.	HM595508.1	Ascomycota	98
33	*Cryptosporiopsis ericae*	HQ889712.1	Ascomycota	100	98	*Phomopsis* sp.	HQ914852.1	Ascomycota	98
34	*Cryptosporiopsis ericae*	JN655660.1	Ascomycota	100	99	*Phomopsis vaccinii*	KC488258.1	Ascomycota	100
35	*Cryptosporiopsis radicicola*	KC311507.1	Ascomycota	100	100	*Plectosphaerella cucumerina*	AB685480.1	Ascomycota	100
36	*Diaporthe cf. nobilis*	KC343146.1	Ascomycota	100	101	*Resinicium bicolor*	DQ826537.1	Basidiomycot	100
37	*Diaporthe eres*	KF017914.1	Ascomycota	100	102	*Rhizopycnis vagum*	KF019248.1	Ascomycota	100
38	*Diaporthe oncostoma*	KC343160.1	Ascomycota	100	103	*Sarea difformis*	FJ903295.1	Ascomycota	100
39	*Dothideomycetes* sp.	JQ905828.1	Ascomycota	95	104	*Septoria provencialis*	DQ303096.1	Ascomycota	100
40	Fungal endophyte sp.	FJ025270.1	Unknown	100	105	*Sphaerulina amelanchier*	KF251634.1	Ascomycota	100
41	Fungal endophyte sp.	FJ466716.1	Unknown	99	106	*Tetracladium* sp.	JN569101.1	Ascomycota	100
42	Fungal sp.	KC506341.1	Unknown	98	107	*Thielavia hyalocarpa*	AB470856.1	Ascomycota	100
43	Fungal sp.	GQ906971.1	Unknown	100	108	*Tricharina ochroleuca*	KF871459.1	Ascomycota	100
44	*Fusarium oxysporum*	HQ696895.1	Ascomycota	100	109	*Trichoderma asperellum*	HM246517.1	Ascomycota	100
45	*Fusarium oxysporum*	KC977497.1	Ascomycota	100	110	*Trichoderma citrinoviride*	KP256793.1	Ascomycota	100
46	*Fusarium proliferatum*	FJ545381.1	Ascomycota	100	111	*Trichoderma erinaceum*	KJ093622.1	Ascomycota	99
47	*Fusarium solani*	KC202941.1	Ascomycota	100	112	*Trichoderma gamsii*	KP715352.1	Ascomycota	99
48	*Fusarium solani*	KF030978.1	Ascomycota	99	113	*Trichoderma hamatum*	KC884769.1	Ascomycota	100
49	*Geomyces pannorum*	AJ509868.1	Ascomycota	100	114	*Trichoderma harzianum*	KC569346.1	Ascomycota	100
50	*Geomyces pannorum*	AJ509869.1	Ascomycota	100	115	*Trichoderma harzianum*	KC847182.1	Ascomycota	99
51	*Geomyces* sp.	JX270359.1	Ascomycota	100	116	*Trichoderma harzianum*	KF053675.1	Ascomycota	100
52	*Glomerella acutata*	AB042300.1	Ascomycota	100	117	*Trichoderma koningiopsis*	KC884814.1	Ascomycota	100
53	*Guignardia mangiferae*	FJ538349.1	Ascomycota	100	118	*Trichoderma* sp.	AY380901.1	Ascomycota	99
54	*Hydnochaete tabacina*	JQ279562.1	Basidiomycota	100	119	*Trichoderma tawa*	KC847191.1	Ascomycota	100
55	*Hypocrea pachybasioides*	JN628070.1	Ascomycota	100	120	*Trichoderma velutinum*	DQ083010.1	Ascomycota	100
56	*Hypocrea virens*	JQ398843.1	Ascomycota	100	121	*Umbelopsis nana*	KC489506.1	Zygomycota	100
57	*Ilyonectria radicicola*	KF240805.1	Ascomycota	100	122	*Umbelopsis nana*	KC816011.1	Zygomycota	100
58	*Irpex* sp.	FJ750850.1	Basidiomycota	100	123	*Umbelopsis ramanniana*	JN198475.1	Zygomycota	100
59	*Lachnum soppittii*	AB481265.1	Ascomycota	97	124	*Umbelopsis* sp.	KF313132.1	Zygomycota	99
60	*Lachnum virgineum*	JF937586.1	Ascomycota	100	125	Uncultured ectomycorrhiza	FJ553509.1	Ascomycota	95
61	*Lecythophora fasciculata*	GQ377492.1	Ascomycota	100	126	Uncultured *Lachnum*	JQ347177.1	Ascomycota	95
62	*Leptodontidium orchidicola*	GU479910.1	Ascomycota	100	127	*Varicosporium elodeae*	JN655610.1	Ascomycota	100
63	*Leptosphaeria microscopica*	KJ572123.1	Ascomycota	98	128	Zygomycete sp.	AY787740.2	Zygomycota	97
64	*Leptosphaeria* sp.	AB752252.1	Ascomycota	99	129	*Zygorhynchus moelleri*	JF327815.1	Zygomycota	100
65	*Leptosphaeria* sp.	AJ608969.1	Ascomycota	99					

Supplementary Table S2. Characteristics and conditions of sampling sites in Korea. All data were collected in 2013.

Locations	A	B	C	D	E	F	G	H
Latitude	36° 51' 55.73" N	38° 4' 29.16" N	36° 22' 0.35" N	36° 59' 26.80" N	36° 24' 44.35" N	37° 14' 3.19" N	37° 35' 42.43" N	37° 22' 23.82" N
Longitude	126° 26' 2.90" E	127° 28' 12.73" E	127° 33' 23.73" E	129° 23' 36.28" E	127° 8' 24.97" E	128° 5' 47.90" E	128° 22' 28.41" E	128° 15' 57.84" E
Altitude (m)	223	449	207	251	389	502	616	509
Mean rainfall (mm)	94.4	23.3	162.6	146.1	151.7	199.2	36	158.2
Mean temperature (°C)	22.2	22.8	23.9	19.3	23.1	23.9	21.5	22.7
pH	5.2	5.4	6.3	6	5.7	6.3	5.4	5.5
Organic matter (g/kg)	23.8	42.9	23.1	20.3	19.8	25.5	27.9	24.8
Available phosphate (mg/kg)	242.8	436	289.9	168.7	309.3	483.7	570.9	506.1
K (cmol+/kg)	1.3	0.6	0.7	3.4	0.5	0.7	0.8	1.2
Ca (cmol+/kg)	1.8	4.7	7.1	4.8	4.1	6.9	4.1	5
Mg (cmol+/kg)	0.6	1.6	1.9	1.8	1	2	1	1.3
Electrical condictivity	0.6	0.4	0.3	0	0.6	0.6	0.7	0.6

Locations	I	J	K	L	M	N	O	P
Latitude	37° 31' 1.93" N	36° 59' 5.25" N	36° 58' 16.11" N	37° 50' 17.47" N	35° 23' 2.00" N	37° 35' 34.97" N	36° 34' 5.94" N	37° 23' 45.24" N
Longitude	128° 55' 38.31" E	127° 38' 40.13" E	128° 55' 16.06" E	128° 5' 5.26" E	127° 55' 15.86" E	127° 21' 41.70" E	128° 8' 40.93" E	127° 31' 45.16" E
Altitude (m)	562	400	558	577	257	271	218	308
Mean rainfall (mm)	112.6	229.9	160	84.6	99.5	87.7	125.7	115.1
Mean temperature (°C)	19	24.6	20.3	23.3	23.1	23.7	22.9	23.3
pH	5.9	5.8	5.6	5.6	4.5	5.9	7.7	5.8
Organic matter (g/kg)	114	17.9	18.3	22.7	23.5	24.6	22.1	20.6
Available phosphate (mg/kg)	721.6	374.8	254.7	450	17	320.2	232.3	300.3
K (cmol+/kg)	4.9	0.4	0.5	0.7	0.9	0.5	0.7	0.4
Ca (cmol+/kg)	7.5	3.6	5	3.9	2.9	5	6.1	3.9
Mg (cmol+/kg)	1.1	1	2	1.1	0.6	1.1	2.1	1.1
Electrical condictivity	0.7	0.4	0.1	0.5	0.3	0.6	0.7	0.7

Locations	Q	R	S	T	U	V	W	X
Latitude	35° 59' 32.19" N	35° 35' 55.03" N	35° 17' 36.97" N	35° 48' 2.35" N	35° 33' 59.93" N	33° 23' 29.13" N	33° 19' 56.85" N	34° 59' 4.86" N
Longitude	128° 42' 42.43" E	127° 38' 56.82" E	127° 21' 21.15" E	127° 18' 46.81" E	126° 54' 53.71" E	126° 19' 57.83" E	126° 22' 36.96" E	126° 29' 45.84" E
Altitude (m)	554	561	216	553	261	235	506	401
Mean rainfall (mm)	63.1	50.9	116.5	60.2	49.6	160.5	156	84.4
Mean temperature (°C)	24.3	22.7	22	21.7	23.5	21.7	22.2	22
pH	5.6	5.7	5.8	5.6	5.3	5.3	5.2	5.6
Organic matter (g/kg)	26.3	28.3	33.5	28.6	30.3	49.7	54.8	17
Available phosphate (mg/kg)	551	269.9	191.2	463.7	193.2	599.8	302	388
K (cmol+/kg)	0.6	0.5	0.5	0.7	1	1.2	1.1	0.9
Ca (cmol+/kg)	5.3	3.9	5	3.9	5.8	4.6	4.4	5.6
Mg (cmol+/kg)	1.5	0.8	1.4	1	1.9	1.8	1.5	1.7
Electrical condictivity	0.7	0.5	0.4	0.4	0.2	1.7	1.2	1.2
